# ORBE II Study: Clinical Characteristics and Outcomes After Treatment with Benralizumab According to Airflow Obstruction Status and Smoking Habit

**DOI:** 10.3390/jcm14227900

**Published:** 2025-11-07

**Authors:** Carlos Martínez-Rivera, Marina Blanco-Aparicio, Francisco Casas-Maldonado, Fernando Sánchez-Toril López, Marta Palop-Cervera, Luis F. Cassini, Jose Luis Sanchez-Trincado, Elisa Luzon, Javier Nuevo, Laia Secall, Marta González-Sierra, Carmen Paula Barragán, Alicia Padilla-Galo

**Affiliations:** 1 Hospital Universitario Germans Trias i Pujol, Carretera de Canyet, S/N, 08916 Badalona, Spain; 2 Hospital Universitario de A Coruña, As Xubias, 84, 15006 A Coruña, Spain; 3 Hospital Universitario Clínico San Cecilio, Av. del Conocimiento, S/N, 18007 Granada, Spain; 4 Hospital Arnau de Vilanova-Lliria, Carrer de Sant Clement, 12, Campanar, 46015 Valencia, Spain; 5 Hospital de Sagunto, Av. Ramon i Cajal, S/N, 46520 Sagunto, Spain; 6 Medical Department, AstraZeneca Farmacéutica S.A, Calle del Puerto de Somport, 21-23, 28050 Madrid, Spain; 7 Hospital Universitario Virgen de la Victoria, Campus de Teatinos, S/N, 29010 Malaga, Spain

**Keywords:** benralizumab, severe eosinophilic asthma, ORBE II, observational, real world, airflow obstruction, smoking

## Abstract

**Background/Objectives**: Evidence on the use of biologics in severe eosinophilic asthma (SEA) associated with persistent airflow obstruction (PAO) status and smoking habits is scarce. As these factors could potentially impact real-world benralizumab clinical effects, this study was conceived to provide a deeper understanding of these specific patient subgroups. **Methods**: This observational, retrospective ORBE II study (NCT04648839) involved 204 adults with uncontrolled SEA treated with benralizumab in Spain. In this analysis, patients were categorized by baseline PAO status (PAO− or PAO+) and smoking habit (SMK− or SMK+) to assess baseline characteristics and clinical outcomes after one year of follow-up. The impact of smoking on PAO+ patients was also analyzed. **Results**: This analysis showed that 75.6% of patients had PAO and 36.9% were former/current smokers. After benralizumab, both PAO subgroups showed substantial improvement, with higher proportions of PAO+ patients achieving prespecified clinical objectives. Benralizumab benefited patients regardless of their smoking habit, though patients who had never smoked exhibited slightly fewer exacerbations, greater improvements in ACT scores, and a higher proportion achieved a ≥100 mL increase in pre-BD FEV_1_. An analysis of the impact of smoking on the PAO+ subgroup showed that while a similar proportion of patients were able to eliminate exacerbations and discontinue OCS use, higher percentages of PAO+SMK− patients achieved an ACT score ≥ 20 and a ≥100 mL increase in pre-BD FEV_1_. **Conclusions**: PAO is common among patients included in ORBE II, and a considerable proportion are former/current smokers. This study showed that clinical outcomes improved after benralizumab initiation regardless of these factors, highlighting its potential as a valuable therapeutic option for managing SEA. These findings also emphasize the need for further real-world evidence to optimize treatment strategies for diverse patient subgroups.

## 1. Introduction

Asthma is a chronic respiratory condition characterized by features such as airflow obstruction, airway inflammation and bronchial hyperresponsiveness. Despite recent therapeutic advances, asthma and, in particular, severe asthma (SA) remains a global problem with a significant burden on healthcare systems and patient welfare (symptoms, exacerbations, treatment-associated side effects, quality of life) [1].

Asthma encompasses a wide spectrum of symptoms and severity levels. While variable and reversible airflow obstruction is a hallmark of asthma, certain patients with long-standing persistent asthma may experience persistent airflow obstruction (PAO). PAO, also known as chronic airflow obstruction and usually defined by a FEV_1_/FVC ratio < 0.7, is a common manifestation in around 16% of asthma patients and increases in prevalence with age, affecting 45% of individuals over the age of 50 [2,3,4]. More than 50% of patients with SA develop PAO, which results from chronic airway inflammation, airway remodeling mechanisms and lung tissue hyperreactivity [5,6]. Eosinophils contribute to airway remodeling and airflow obstruction—via epithelial injury, smooth muscle hypertrophy, defective tissue repair mechanisms—and to mucus hypersecretion in T2 asthma [7,8]. Indeed, higher blood or sputum eosinophil counts confer a 2- to 4-fold increased risk of PAO [9,10], and PAO is associated with a more severe disease, decreased quality of life, and heightened healthcare resource utilization, making PAO+ asthma particularly challenging to manage [2,11].

Smoking is a major contributor to airway damage and obstruction [2]. Active smoking could be associated with airway inflammation and mucus production, and higher incidence, severity and mortality in asthma [12]; 20–35% of people with asthma are active smokers—similar to the general population [13]—yet prevalence falls to ~1–8% in SA [14,15], and these patients are frequently underrepresented or excluded from randomized trials of asthma-targeted therapies [16,17,18].

The development of biological therapeutics has ushered in the era of precision medicine for the management of SA, an advancement that is propelling clinical goals toward disease remission and offering new hope for refractory patients. Benralizumab, an interleukin-5 receptor alpha subunit-directed cytolytic monoclonal antibody for patients with severe eosinophilic asthma (SEA), depletes blood and airway tissue eosinophils, significantly reducing exacerbations and improving lung function and asthma symptoms [19,20]. In the real-world context, studies such as ORBE II have provided further evidence on the clinical effects of benralizumab in adult patients with SEA [21]. In relation to airflow obstruction, a post hoc pooled analysis of the SIROCCO and CALIMA trials showed that the effectiveness of benralizumab was independent of PAO status, with even better outcomes observed in PAO+ patients [22]. Evidence on the impact of smoking habits on benralizumab response, however, remains very limited. Therefore, additional real-world evidence is needed to evaluate the beneficial effects of benralizumab in these patient profiles.

In this retrospective subgroup analysis of the ORBE II population, we aimed to characterize the baseline clinical characteristics of SEA patients according to PAO status and smoking habits, and to describe the clinical benefits of benralizumab for patients grouped within these subcategories.

## 2. Materials and Methods

### 2.1. Study Design

The ORBE II study (NCT04648839) is a multicenter, observational, retrospective research whose design has been previously described [21]. In brief, 204 uncontrolled SEA adult patients who were administered benralizumab according to routine clinical practice were included in the ORBE II study. Data were obtained from the electronic medical records of the 15 participating asthma units (February and July 2021). The study’s main objective was to characterize baseline characteristics and treatment patterns of SEA patients receiving benralizumab.

In this analysis, for PAO and smoking status effects on clinical outcomes in patients under benralizumab treatment, ORBE II patients were classified by baseline airflow obstruction status (PAO− and PAO+) and by smoking habits (SMK− and SMK+); within the PAO+ subgroup, outcomes were also compared by smoking habits. We evaluated the sociodemographic and clinical profiles of these patients at baseline and assessed their clinical outcomes up to one year after benralizumab treatment initiation.

As a measurement of benralizumab beneficial effects the results corresponding to the first 12 months of follow-up were presented. We considered four main clinical objectives, including the absence of exacerbations, no need for maintenance oral corticosteroids (mOCS), controlled symptoms (asthma control test [ACT] score ≥ 20), and a pre-bronchodilator (BD) FEV_1_ improvement of ≥100 mL [21].

### 2.2. Patient Classification

In this analysis, airflow obstruction status was defined as persistent if baseline post-BD FEV_1_/FVC index was <0.7. Patients were classified into two groups, those with baseline persistent airflow obstruction (PAO+) or without (PAO−). Patients were also classified according to their smoking habit at baseline as smokers (former/current) (SMK+) or non-smokers (SMK−). Grouping former and current smokers was planned to capture the overall impact of tobacco exposure—including potential long-term effects after cessation –and because the low number of current smokers in SA limits the stability of separate estimates.

### 2.3. Statistical Analysis

The descriptive analysis of sociodemographic and clinical outcomes was conducted according to the criteria established for the total sample of the ORBE II study [21]. Analyses were performed using R software, v. 4.3.0 (www.R-project.org, accessed on 19 March 2024), and its “dplyr”, “gtsummary” and “psych” packages.

## 3. Results

### 3.1. ORBE II Cohort Classification Based on Baseline PAO and/or Smoking Habit

**Figure 1:**
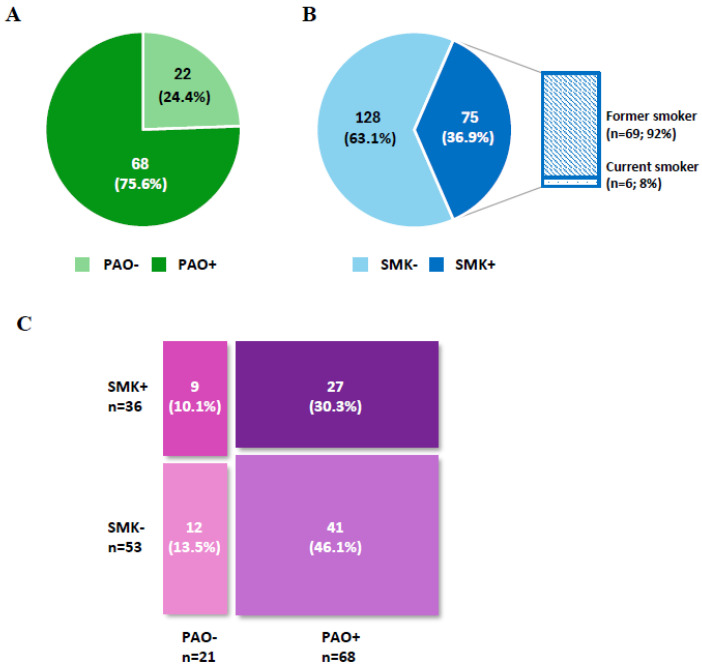
Distribution of patients regarding (**A**) persistent airflow obstruction (PAO+/PAO−) (n = 90) and (**B**) smoking status (SMK+/SMK−) (n = 203) at baseline. (**C**) Bidimensional representation of both characteristics (n = 89).

In the overall population of SEA patients in the ORBE II study (N = 204), baseline data to identify PAO status or smoking habits were available for a total of 90 (44.1%) and 203 (99.5%) patients, respectively (Figure 1A,B). Considering only patients with available PAO data, 22 (24.4%) were PAO− and 68 (75.6%) were PAO+. In patients with available data about their smoking habit, 128 (63.1%) were never-smokers (SMK−) and 75 (36.9%) were former or current smokers (SMK+). Within the SMK+ subgroup, 92% were former smokers and 8% smoked at baseline, indicating that 2.9% of the patient population with available data were active smokers.

Information at baseline for both conditions was available from 89 patients (43.6%) (Figure 1C). The bidimensional PAO/SMK distribution showed that the most prevalent group was PAO+SMK−. The proportion of PAO+ patients was 75.0% among smokers and 77.4% among non-smokers, while the proportion of smokers within the PAO− and PAO+ subgroups was 42.9% and 39.7%, respectively.

### 3.2. Baseline Characterization of Patient Subgroups

The two PAO−based patient subgroups exhibited similar baseline characteristics, including a similar age at asthma onset and asthma duration and comparable proportion of patients with no previous biologic treatment (Table 1). Despite the similar percentage of smokers, the median number of pack-years was apparently higher for PAO− than for PAO+ patients (Table 1). Concerning comorbidities, fewer PAO+ patients presented chronic rhinosinusitis with nasal polyps (CRSwNP) (32.4% vs. 45.5%) and obstructive sleep apnea syndrome (OSAS) (11.8% vs. 22.7%) but 5.9% of them also had COPD. Independently of PAO status, most patients presented severe exacerbations (SE) at baseline, with this rate even higher among PAO− patients, who also needed more frequent hospitalizations and emergency department (ED) visits. Additionally, 19.4% of the PAO+ individuals showed OCS dependency, a percentage that rose to 42.1% in the PAO− population. Notably, OCS-dependent PAO− patients used a remarkably high median (IQR) OCS daily dose (29.0 [14.2–39.0] mg), whereas PAO+ patients reported less than half of that dose (13.0 [9.4–20.2] mg). With regard to asthma control, nearly 80% of patients had an ACT score < 20 regardless of their obstructive baseline condition. For lung function, the proportion of patients with a pre-BD FEV_1_ < 80% was 91.0% in PAO+ and 54.5% in PAO− subgroups, along with a mean (SD) pre-BD absolute FEV_1_ of 1625.4 (624.3) mL in PAO+ and 2251.4 (847.9) in PAO−, respectively.

When we assessed the features of patients according to smoking habit at baseline, we observed that females were predominant among SMK− patients (69.5%) but not among smokers (49.3%) (Table 1). The SMK+ subgroup presented a shorter asthma duration with a similar age at onset, and a slightly higher proportion of patients had allergic asthma. They had a median (IQR) of 10 (7.0–24.5) pack-years and included a particularly high proportion of obesity (36.8%), comorbid COPD (17.3%), OSAS (21.3%), and bronchiectasis (17.3%). Both subgroups reported similar levels of SE, hospitalizations and ED visits. While the SMK− subgroup included a slightly higher proportion of OCS-dependent patients (30.4% versus 25.8%), a higher median OCS daily dose was observed in SMK+ patients. Asthma control and lung function were similarly impaired in both subgroups, with approximately 83% of patients having an ACT of ≤20 and around 70% presenting a pre-BD FEV_1_ < 80%.

### 3.3. Benralizumab Response According to PAO Status or Smoking Habit

**Figure 2:**
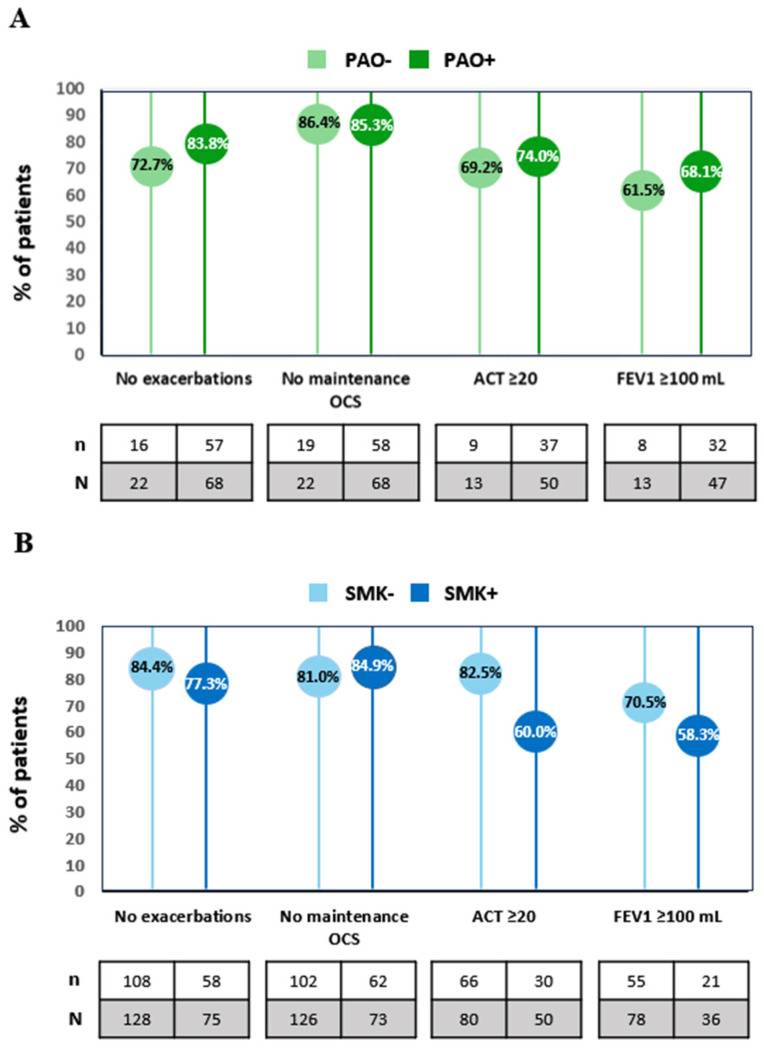
Proportion of patients achieving clinical objectives after benralizumab treatment according to (**A**) persistent airflow obstruction (PAO+/PAO−) or (**B**) smoking status (SMK+/SMK−). ACT, asthma control test; FEV_1_ = Forced Expiratory Volume in one second; OCS, oral corticosteroids; PAO, persistent airflow obstruction; SMK, smoker.

Based on PAO status at baseline, both subgroups showed a remarkable reduction in the number of SE, the frequency of hospitalizations and ED visits, achieving similar trends and ranging from 88.9% to 100% between subgroups after up to one year of follow-up (Table 2). A reduction in OCS use was observed in both subgroups, with 87.5% of PAO− patients reaching a reduction of ≥50% in OCS use and 75% achieving complete withdrawal, along with a similar increase in the ACT score. Although both subgroups showed improvements in lung function, PAO+ subjects obtained a more pronounced mean (SD) increase of 402.6 (427.9) mL, but their final volume remained slightly lower than that of the PAO− subgroup at the end of follow-up (Table 2). In general, the PAO+ subgroup attained a higher percentage of patients achieving clinical objectives. Specifically, 83.8% of PAO+ individuals reached a total elimination of SE, 74.0% an ACT score ≥ 20, and 68.1% a ≥100 mL improvement in pre-BD FEV_1_. Nonetheless, the proportion of patients who did not use maintenance OCS use was similar in both subgroups (Figure 2A). Notably, FeNO was only reduced in PAO− patients after one year of follow-up with a median reduction of 12 ppb.

All patients, regardless of their smoking habit, seemed to benefit from benralizumab treatment; however, it is noteworthy that non-smokers showed a higher percentage of reduction in SE, reaching a 92.0% decrease in SMK− subgroup and 84.0% in SMK+ subgroup (Table 2). The same pattern was observed in ACT score, where the gain in mean score and the percentage of patients with ACT increase ≥ 3 was slightly higher in non-smokers, although both subgroups showed similar rates of OCS reduction. At a functional level, both subgroups achieved a similar mean pre-BD FEV_1_ volume, although the mean (SD) predicted pre-BD FEV_1_ (%) after one year was lower among smokers (81.9 [22.4] for SMK− and 72.5 [20.7] for SMK+). In fact, the improvements from baseline were better in the non-smokers group. The mean (SD) increment in pre-BD FEV_1_ for SMK− patients was 355.3 (426.8) mL with 50.0% of patients reaching an improvement of ≥230 mL, whereas for SMK+ patients, the increase was 278.3 (380.6) mL, with 41.7% achieving an improvement of ≥230 mL. Similar results were obtained for the clinical objectives regarding exacerbation and OCS use elimination in both subgroups; however, the percentage of patients that achieved an ACT ≥ 20 and pre-BD FEV_1_ ≥ 100 mL was superior among non-smokers. Notably, 82.5% of SMK− and 60% of SMK+ scored an ACT ≥ 20, and 70.5% of SMK− and 58.3% of SMK+ showed a ≥100 mL increase in pre-BD FEV_1_ (Figure 2B). A greater reduction in FeNO values compared to baseline was observed in non-smokers (Table 2).

### 3.4. Exploring the Role of Smoking in PAO+ Patients

PAO+ patients (N = 68) were also characterized according to their smoking habits. Their baseline characteristics are summarized in Supplementary Table S1. The majority of PAO+SMK− subgroup patients were females. Allergic asthma prevalence was 39.0% among PAO+SMK− patients and 25.9% among PAO+SMK+ patients, with the latter subgroup showing a higher prevalence of COPD and bronchiectasis. Peripheral blood eosinophil counts (BEC) were notably elevated in PAO+SMK− patients, but both subgroups had similar median levels of IgE and FeNO. In general, PAO+SMK− patients presented at baseline a higher number of SE, hospitalizations and ED visits. Furthermore, a notably high number of PAO+SMK− patients had poor asthma control, with 85.7% of patients having an ACT < 20 and a markedly impaired lung function with a mean (SD) pre-BD FEV_1_ of 1439.0 (452.8) mL.

After one year of follow-up, both subgroups showed similar reductions in the number of SE (Table 3). Nevertheless, the proportion of patients showing SE reductions was slightly higher in the PAO+SMK− subgroup (97.0%). Both subgroups also presented an identical percentage reduction in hospitalizations (100% in each subgroup), and large reductions in the rate of ED visits, slightly more pronounced in the non-smoking group (85.7% in PAO+SMK− and 75.5% in PAO+SMK+). Regarding asthma control, PAO+SMK− patients achieved a markedly higher mean ACT score with a mean (SD) increase of 7.5 (6.6) points and of 4.9 (5.5) in PAO+SMK+ patients. Both subgroups achieved improvements in lung function, with increases in mean [SD] pre-BD FEV_1_ volumes of 454.0 [462.5] mL for PAO+SMK− and 311.8 [353.6] mL for PAO+SMK+.

**Figure 3:**
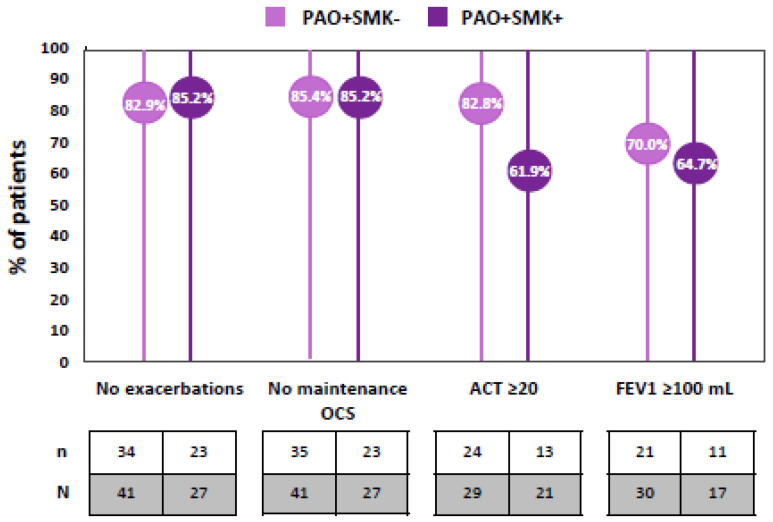
Attainment of clinical objectives after benralizumab treatment in patients with persistent airflow obstruction (PAO+) according to their smoking habit (SMK−/+). ACT, asthma control test; FEV_1_ = Forced Expiratory Volume in one second; OCS, oral corticosteroids; PAO, persistent airflow obstruction; SMK, smoker.

In terms of the pre-defined clinical objectives following benralizumab treatment, a large number of PAO+ patients achieved zero exacerbations and did not require mOCS, irrespective of their smoking habit (Figure 3). However, despite beneficial outcomes regarding asthma control in both subgroups, a higher percentage of PAO+SMK− patients achieved an ACT score ≥ 20 and a pre-BD FEV_1_ (mL) increase ≥ 100 mL (Figure 3).

## 4. Discussion

The findings of this subgroup analysis of the ORBE II study underscore the clinical benefits of benralizumab in SEA patients, regardless of their PAO status or smoking history. Notably, PAO+ patients constitute a significant portion of asthmatic patients—75.6% of participants in this study. This is a higher proportion than in the post hoc pooled analysis of the SIROCCO and CALIMA studies, in which 63% of patients presented PAO [22], but suggests nonetheless that PAO might be a predominant feature among SEA patients, particularly those selected to receive a biological treatment in the real-world setting. Furthermore, 36.9% were former/current smokers, a considerable subpopulation that is generally excluded in clinical trials assessing biological drug effectivity for SA.

It should be emphasized that the proportion of smokers among PAO+ and PAO− was similar, suggesting that PAO+ patients may be suffering from persistent obstruction originating primarily from other reasons, possibly driven by underlying eosinophilic inflammation. Indeed, blood and sputum eosinophilia correlate with more severe airflow obstruction [23,24]. Interestingly, certain sputum biomarkers associated with sputum eosinophilia, such as periostin, can identify SA patients with PAO [25]. Nevertheless, while PAO and smoking are closely associated with COPD, it is crucial that both diseases are accurately diagnosed, given the risk of misclassifying patients who may have asthma or both conditions as solely having COPD [26].

The detailed analysis of PAO subgroups revealed that in this study PAO+ patients, who typically present a more severe disease phenotype [11], showed poorer baseline lung function along with lower mean SE and hospitalizations and a higher mean OCS dose at baseline than their PAO− counterparts. In turn, PAO+ subgroup generally presented substantially greater clinical improvements with benralizumab. Indeed, although both PAO− and PAO+ patients achieved practically the same pre-BD FEV_1_ global volumes after treatment, PAO+ patients demonstrated a 3-fold increase compared to PAO− patients (Table 2). These findings are consistent with previous clinical studies in which improvements were observed for both populations and also especially for the PAO+ subgroup, suggesting that addressing eosinophilic inflammation could be beneficial for improving the persistent obstruction and remodeling associated with SA [22]. Other studies have also shown that benralizumab can ameliorate other functional impairments, such as lung hyperinflation or airway resistance and hyperresponsiveness [27,28,29]. Altogether, targeting eosinophils through benralizumab-mediated IL-5R inhibition reduces mucus, air trapping and airway inflammation [30,31].

Smoking has been identified as a major risk factor for SA and irreversible airway changes. Specifically, nicotine not only affects neutrophil function, contributing directly to the pathogenesis of chronic inflammatory disorders, but also has an impact on eosinophilic activation [32]. In fact, a recent study demonstrated that a history of smoking may drive eosinophilic airway activation and airway inflammation [33]. Analyzing the ORBE II population by smoking habit revealed two subgroups with similar asthma control and lung function. However, the SMK+ population exhibited a higher proportion of patients with obesity and respiratory comorbidities, reinforcing the fact that smokers are a complex population, which could in turn contribute to a putative asthma misdiagnosis. In this sub-analysis, benralizumab showed favorable responses in both smokers and non-smokers. In fact, other studies have also demonstrated that the effectiveness of different biologics was similar in both never-smokers and former smokers with SA, indicating that a previous history of smoking does not rule out a clinical benefit from biological therapy [15].

The greatest difference in baseline values between PAO−/+ patients was in lung function impairment, and as expected, a low baseline pre-BD FEV_1_ was a particular finding in PAO+ individuals. In contrast, a more similar baseline lung function was observed between smokers and never-smokers. Nevertheless, a higher percentage of PAO+ patients achieved clinical objectives, highlighting the fact that the airway eosinophil clearance mediated by benralizumab treatment can highly benefit PAO+ patients, despite more impaired lung function at baseline. This reinforces the potential of targeted biological therapies for addressing SEA in more complex patient profiles [34].

We also investigated whether smoking, as a potential pathophysiological trigger for obstruction, could influence clinical outcomes with benralizumab in PAO+ patients. To our knowledge, this is the first study that investigates benralizumab clinical effects in PAO+SMK+ patients. Baseline characteristics of PAO+SMK− and PAO+SMK+ populations reflected a higher percentage of comorbidities in PAO+SMK+ individuals, with a notably high proportion of individuals with CRSwNP, COPD and bronchiectasis. This may possibly result in a more convoluted clinical picture and a higher overall disease burden in these patients. However, PAO+SMK− patients had a slightly lower pre-BD FEV_1_ than their counterparts and a high prevalence of poor asthma control, indicating that smoking is not the only trigger for impaired respiratory function. In terms of pre-defined clinical objectives after benralizumab, while PAO+ patients similarly resolved exacerbations and eliminated OCS use independently of their smoking habit, slightly fewer smokers achieved asthma control (ACT ≥ 20) and lung function (pre-BD FEV_1_ ≥ 100 mL) objectives, suggesting that obstruction caused by smoking may be more difficult to treat. Further studies are needed to better characterize these patients and to determine whether smoking cessation strategies may help ever-smokers with SA achieve optimal inflammation and symptom control.

The observational retrospective nature of our study and the absence of a control arm present inherent limitations. Because of the size of the subgroups, some findings—particularly those extracted from the analysis of the PAO/SMK bidimensional categorization of patients—need to be interpreted cautiously. Furthermore, due to the limited number of active smokers in this study, we could not perform any specific analysis to discriminate them from former smokers, so possible differences between them may have been missed. Lastly, the study design precluded formal comparisons between subgroups.

## 5. Conclusions

This subgroup analysis of the ORBE II study provides solid real-world evidence of the beneficial effects of benralizumab in improving clinical outcomes in SEA patients, regardless of PAO status or smoking history. These results support the pivotal role of eosinophilic inflammation and the broader benefits of benralizumab for SEA patients, particularly in subgroups that traditionally face poorer prognoses and fewer treatment options.

## Figures and Tables

**Table 1:** Baseline sociodemographic and clinical characteristics of ORBE II patients classified by PAO status (n = 90) or smoking habit (n = 203).

Variables	PAO− n = 22	PAO+ n = 68	SMK− n = 128	SMK+ n = 75
**Sex, n (%)**				
Female Male	15 (68.2%) 7 (31.8%)	42 (61.8%) 26 (38.2%)	89 (69.5%) 39 (30.5%)	37 (49.3%) 38 (50.7%)
**Age (years)**				
Mean (SD)	52.5 (11.4)	55.3 (13.3)	56.7 (13.3)	56.3 (10.2)
**BMI (Kg/m^2^); N ^a^**	22	68	119	68
Mean (SD)	27.5 (6.7)	28.2 (6.5)	27.6 (6.2)	29.1 (6.5)
Obese ^b^, n (%)	6 (27.3%)	22 (32.4%)	29 (24.4%)	25 (36.8%)
**Age at asthma onset (years); N ^a^**	17	51	91	54
Mean (SD)	32.3 (15.8)	32.2 (18.1)	34.6 (16.5)	34.5 (16.2)
**Asthma duration (years) ^c^; N ^a^**	17	51	35	31
Mean (SD)	19.5 (14.0)	20.6 (14.2)	24.5 (13.3)	18.9 (13.6)
**Allergic asthma, n (%)**	6 (27.3%)	23 (33.8%)	41 (32.0%)	27 (36.0%)
**Smoking history, n (%); N ^a^**	21	68	52	36
Never smoker	12 (57.1%)	41 (60.3%)	128 (100.0%)	0 (0.0%)
Former smoker	9 (42.9%)	24 (35.3%)	0 (0.0%)	69 (92.0%)
Smoker	0 (0.0%)	3 (4.4%)	0 (0.0%)	6 (8.0%)
**Cigarette pack-year, n; N ^a^** Median (IQR)	7 20 (15.6–26.3)	19 10 (5.8–14.3)	0 NA	57 10 (7.0, 24.5)
**Comorbidities, n (%) ^d^**	22 (100.0%)	60 (88.2%)	120 (93.8%)	70 (93.3%)
CRSwNP COPD Bronchiectasis	10 (45.5%) 0 (0.0%) 0 (0.0%)	22 (32.4%) 4 (5.9%) 4 (5.9%)	50 (39.1%) 1 (0.8%) 1 (0.8%)	25 (33.3%) 13 (17.3%) 13 (17.3%)
GERD	3 (13.6%)	14 (20.6%)	25 (19.5%)	17 (22.7%)
Osteoporosis	3 (13.6%)	7 (10.3%)	25 (19.5%)	7 (9.3%)
OSAS	5 (22.7%)	8 (11.8%)	11 (8.6%)	16 (21.3%)
HBP	4 (18.2%)	12 (17.6%)	23 (18.0%)	9 (12.0%)
Diabetes	1 (4.5%)	7 (10.3%)	11 (8.6%)	7 (9.3%)
Depression	0 (0.0%)	6 (8.8%)	14 (10.9%)	3 (4.0%)
Cataracts	0 (0.0%)	2 (2.9%)	3 (2.3%)	3 (4.0%)
**Patients with prior biologic treatment, n (%) ^d^**				
Omalizumab	4 (18.2%)	9 (13.2%)	18 (14.1%)	14 (18.7%)
Mepolizumab **^d^**	2 (9.1%)	12 (17.6%)	21 (16.4%)	12 (16.2%)
Reslizumab	0 (0.0%)	2 (2.9%)	3 (2.4%)	2 (2.7%)
Patients with no prior biologic treatment	16 (72.7%)	48 (70.6%)	87 (68.0%)	51 (68.0%)
**Peripheral BEC (cell/µL); N ^a^**	20	65	124	72
Median (IQR)	550 (275.0, 902.5)	500 (200.0, 730.0)	530 (200.0, 800.0)	440 (240.0, 685.0)
**Total serum IgE concentration, (IU/mL); N ^a^**	18	52	97	46
Median (IQR)	169 (55.7, 434.5)	241 (89.2, 456.3)	128 (44.3, 417.0)	283 (92.8, 465.8)
**FeNO (ppb); N ^a^**	14	48	52	36
Median (IQR)	37 (21.8, 81.2)	39 (19.3, 66.0)	36 (19.9, 61.5)	38 (18.8, 66.3)
**OCS dependency**				
OCS-dependent patients, n/N ^a^ (%)	8/19 (42.1%)	12/62 (19.4%)	35/115 (30.4%)	17/66 (25.8%)
**Daily dose of OCS (mg); N ^a^**	8	12	35	17
Median (IQR)	29 (14.2, 39.0)	13 (9.4, 20.2)	12 (5.0, 24.4)	20 (10.0, 30.0)
Patients with daily OCS dose ≥ 5 mg, n (%)	8 (100.0%)	12 (100.0%)	32 (91.4%)	17 (100.0%)
**Severe exacerbations; N ^a^**	22	68	128	75
Patients with severe exacerbations, n (%)	21 (95.5%)	55 (80.9%)	107 (83.6%)	65 (86.7%)
Severe exacerbations, mean (SD)	3.4 (2.1)	2.5 (2.3)	2.5 (2.5)	2.5 (1.9)
**ED visits; N ^a^**	22	68	128	75
Patients with ED visits, n (%)	9 (40.9%)	21 (30.9%)	43 (33.6%)	28 (37.3%)
ED visits, mean (SD)	0.7 (1.0)	0.6 (1.4)	0.6 (1.8)	0.8 (1.4)
**Hospitalizations; N ^a^**	22	68	128	75
Patients with hospitalizations, n (%)	9 (40.9%)	12 (17.6%)	28 (21.9%)	15 (20.0%)
Hospitalizations, mean (SD)	0.6 (0.8)	0.3 (0.7)	0.3 (0.8)	0.4 (0.9)
**Asthma control; N ^a^**	17	61	47	27
ACT score, mean (SD)	14.4 (4.9)	14.5 (5.5)	14.2 (4.9)	14.1 (5.5)
Patients with ACT score < 20, n (%)	14 (82.4%)	48 (78.7%)	77 (83.7%)	45 (83.3%)
**Lung function; N ^a^**	22	67	98	55
Pre-BD FEV_1_ (mL), mean (SD)	2251.4 (847.9)	1625.4 (624.3)	1836.1 (801.3)	2029.6 (765.1)
Pre-BD FEV_1_ (% predicted), mean (SD)	77.4 (20.2)	56.5 (15.3)	68.6 (21.5)	65.0 (20.4)
Patients with pre-BD FEV_1_ < 80%, n/N ^a^ (%)	12/22 (54.5%)	61/67 (91.0%)	74/106 (69.8%)	45/63 (71.4%)

All values were calculated based on the total of patients with available data (excluding missing values). Proportion of patients within each subgroup was calculated using the total number of patients with available obstruction status and smoking habit data. Airflow obstruction status was defined as persistent (PAO+) if baseline post-BD FEV_1_/FVC index was <0.7. Patients were also classified regarding their smoking habit at baseline into smokers (former/current) (SMK+) or non-smokers (SMK−). ^a^ Patients with available data. ^b^ BMI ≥ 30 Kg/m^2^. ^c^ Time in years since first asthma symptoms occurred. ^d^ Multiple response variable. ACT, asthma control test; BEC, blood eosinophil count; BMI, body mass index; COPD, chronic obstructive pulmonary disease; CRSwNP, chronic rhinosinusitis with nasal polyposis; ED, emergency department; FeNO, fraction of exhaled nitric oxide; FEV_1_, forced expiratory volume in the first second; GERD, gastroesophageal reflux disease; HBP, high blood pressure; IQR, interquartile range; OCS, oral corticosteroids; OSAS, obstructive sleep apnea syndrome; PAO, persistent airflow obstruction, pre-BD, pre-bronchodilator; SD, standard deviation; SMK, smoker.

**Table 2:** Clinical outcomes of ORBE II patient subgroups classified by PAO status and smoking habit at 1-year of follow-up after benralizumab initiation.

Variables	PAO− n = 22	PAO+ n = 68	SMK− n = 128	SMK+ n = 75
**Severe exacerbations; N ^a^**	22	68	128	75
***Baseline***				
Severe exacerbations, mean (SD)	3.4 (2.1)	2.5 (2.3)	2.5 (2.5)	2.5 (1.9)
Patients with zero exacerbations, n (%)	1 (4.5%)	13 (19.1%)	21 (16.4%)	10 (13.3%)
***1-year FUP***				
Severe exacerbations, mean (SD)	0.5 (0.9)	0.3 (0.8)	0.2 (0.7)	0.4 (0.9)
Patients with zero exacerbations, n (%)	16 (72.7%)	57 (83.8%)	108 (84.4%)	58 (77.3%)
**Patients with severe exacerbations reduction, n (%) ^b^**	19 (90.5%)	52 (94.5%)	104 (97.2%)	57 (87.7%)
**Percentage reduction in severe exacerbations**	85.3%	88.0%	92.0%	84.0%
**ED visits; N ^a^**	22	68	128	75
***Baseline***				
ED visits, mean (SD)	0.7 (1.0)	0.6 (1.4)	0.6 (1.8)	0.8 (1.4)
Patients with zero ED visits, n (%)	13 (59.1%)	47 (69.1%)	85 (66.4%)	47 (62.7%)
***1-year FUP***				
ED visits, mean (SD)	0.1 (0.3)	0.1 (0.4)	0.1 (0.3)	0.1 (0.5)
Patients with zero ED visits, n (%)	20 (90.9%)	65 (95.6%)	122 (95.3%)	68 (90.7%)
**Patients with reduction in ED visits, n (%) ^c^**	9 (100.0%)	20 (95.2%)	42 (97.7%)	24 (85.7%)
**Percentage reduction in ED visits**	85.7%	83.3%	83.3%	87.5%
**Hospitalizations; N ^a^**	22	68	128	75
***Baseline***				
Hospitalizations, mean (SD)	0.6 (0.8)	0.3 (0.7)	0.3 (0.8)	0.4 (0.9)
Patients with zero hospitalizations, n (%)	13 (59.1%)	56 (82.4%)	100 (78.1%)	60 (80.0%)
***1-year FUP***				
Hospitalizations, mean (SD)	0.0 (0.2)	0.0 (0.2)	0.1 (0.3)	0.1 (0.3)
Patients with zero hospitalizations, n (%)	21 (95.5%)	66 (97.1%)	123 (96.1%)	72 (96.0%)
**Patients with reduction in hospitalizations, n (%) ^d^**	8 (88.9%)	11 (91.7%)	26 (92.9%)	14 (93.3%)
**Percentage reduction in hospitalizations**	100.0%	100.0%	66.7%	75.0%
**OCS dependency**				
***Baseline***				
OCS-dependent patients, n/N ^a^ (%)	8/19 (42.1%)	12/62 (19.4%)	35/115 (30.4%)	17/66 (25.8%)
***1-year FUP***				
OCS-dependent patients, n/N ^a^ (%)	3/22 (13.6%)	10/68 (14.7%)	24/126 (19.0%)	11/73 (15.1%)
**Daily dose of OCS (mg); N ^a^**	8	12	35	17
***Baseline***				
Median (IQR)	29 (14.2, 39.0)	13 (9.4, 20.2)	12 (5.0, 24.4)	20 (10.0, 30.0)
***1-year FUP***				
Median (IQR)	0 (0.0, 1.3)	3 (0.0, 6.9)	0 (0.0, 6.9)	0 (0.0, 11.5)
**Patients achieving OCS dose reduction ≥ 50%, n (%) ^b^**	7 (87.5%)	8 (66.7%)	22 (62.9%)	10 (58.8%)
**Patients achieving complete OCS withdrawal, n (%)**	6 (75.0%)	6 (50.0%)	18 (51.4%)	10 (58.8%)
**ACT score**				
***Baseline***				
ACT score, mean (SD)	14.4 (4.9)	14.5 (5.5)	14.2 (4.9)	14.1 (5.5)
Patients with ACT score < 20, n/N ^a^ (%)	14/17 (82.4%)	48/61 (78.7%)	77/92 (83.7%)	45/54 (83.3%)
***1-year FUP***				
ACT score, mean (SD)	20.5 (5.5)	21.4 (4.1)	21.9 (4.0)	19.4 (5.5)
Patients with ACT score <20, n/N ^a^ (%)	4 (30.8%)	13 (26.0%)	14 (17.5%)	20 (40.0%)
**Increase in ACT score, mean (SD)**	5.7 (5.5)	6.3 (6.2)	7.3 (6.1)	5.5 (5.8)
**Patients with ACT increase ≥ 3, n/N ^a^ (%)**	6/10 (60.0%)	32/45 (71.1%)	48/64 (75.0%)	27/40 (67.5%)
**Lung function**				
***Baseline;* N ^a^**	22	67	106	63
Pre-BD FEV_1_ (% predicted), mean (SD)	77.4 (20.2)	56.5 (15.3)	68.6 (21.5)	65.0 (20.4)
Patients with pre-BD FEV_1_ < 80%, n (%)	12 (54.5%)	61 (91.0%)	74 (69.8%)	45 (71.4%)
***1-year FUP;* N ^a^**	18	5	36	27
Pre-BD FEV_1_ (% predicted), mean (SD)	88.1 (17.0)	72.2 (18.0)	81.9 (22.4)	72.5 (20.7)
Patients with pre-BD FEV_1_ < 80%, n (%)	3 (23.1%)	32 (68.1%)	43 (48.3%)	29 (64.4%)
***Baseline;* N ^a^**	22	67	98	55
Pre-BD FEV_1_ (mL), mean (SD)	2251.4 (847.9)	1625.4 (624.3)	1836.1 (801.3)	2029.6 (765.1)
***1-year FUP;* N ^a^**	18	5	85	42
Pre-BD FEV_1_ (mL), mean (SD)	2360.8 (705.3)	2079.8 (769.8)	2195.8 (851.5)	2167.6 (724.2)
**Increase in pre-BD FEV_1_ (mL), mean (SD)**	126.9 (292.9)	402.6 (427.9)	355.3 (426.8)	278.3 (380.6)
**Patients with pre-BD FEV_1_ increment ≥ 100 mL, n (%)**	8/13 (61.5%)	32/47 (68.1%)	55/78 (70.5%)	21/36 (58.3%)
**Patients with pre-BD FEV_1_ increment ≥ 230 mL, n (%)**	5/13 (38.5%)	26/47 (55.3%)	39/78 (50.0%)	15/36 (41.7%)
**Patients with pre-BD FEV_1_ increment ≥ 500 mL, n (%)**	0/13 (0.0%)	18/47 (38.3%)	27/78 (34.6%)	12/36 (33.3%)
**FeNO (ppb); N ^a^**	14	48	76	44
***Baseline***				
Median (IQR)	37 (21.8, 81.2)	39 (19.3, 66.0)	36 (19.9, 61.5)	38 (18.8, 66.3)
***1-year FUP***				
Median (IQR)	25 (19.0, 52.9)	44 (18.0, 68.0)	23 (15.4, 59.0)	34 (17.0, 54.0)

All values were calculated based on the total of patients with available data (excluding missing values). ^a^ Patients with available data. ^b^ Proportion of patients with 1 or more exacerbations at baseline who achieved a decrease in the frequency of exacerbations at 1-year FUP. ^c,d^ Calculations were made using the total number of patients with ED visits or hospitalizations, respectively. ACT, asthma control test; BEC, blood eosinophil count; ED, emergency department; FeNO, fraction of exhaled nitric oxide; FEV_1_, forced expiratory volume in the first second; FUP, follow-up; PAO, persistent airflow obstruction; pre-BD, pre-bronchodilator; SD, standard deviation; SMK, smoker.

**Table 3:** Evolution of PAO+ patients based on their smoking habit after 1-year of follow-up with benralizumab (N = 68).

Variables	PAO+	
	SMK− n = 41	SMK+ n = 27
**Severe exacerbations; N ^a^**	41	27
***Baseline***		
Severe exacerbations, mean (SD)	2.9 (2.8)	2.0 (1.4)
Patients with zero exacerbations, n (%)	8 (19.5%)	5 (18.5%)
***1-year FUP***		
Severe exacerbations, mean (SD)	0.3 (0.8)	0.3 (0.7)
Patients with zero exacerbations, n (%)	34 (82.9%)	23 (85.2%)
**Patients with severe exacerbations reduction, n (%) ^b^**	32 (97.0%)	20 (90.9%)
**Percentage reduction in severe exacerbations**	89.7%	85.0%
**ED visits; N ^a^**	41	27
***Baseline***		
ED visits, mean (SD)	0.7 (1.7)	0.4 (0.8)
Patients with zero ED visits, n (%)	27 (65.9%)	20 (74.1%)
***1-year FUP***		
ED visits, mean (SD)	0.1 (0.3)	0.1 (0.6)
Patients with zero ED visits, n (%)	39 (95.1%)	26 (96.3%)
**Patients with reduction in ED visits, n (%) ^c^**	14 (100.0%)	6 (85.7%)
**Percentage reduction in ED visits**	85.7%	75.0%
**Hospitalizations; N ^a^**	128	75
***Baseline***		
Hospitalizations, mean (SD)	0.4 (0.9)	0.1 (0.3)
Patients with zero hospitalizations, n (%)	32 (78.0%)	24 (88.9%)
***1-year FUP***		
Hospitalizations, mean (SD)	0.0 (0.2)	0.0 (0.2)
Patients with zero hospitalizations, n (%)	40 (97.6%)	26 (96.3%)
**Patients with reduction in hospitalizations, n (%) ^d^**	9 (100.0%)	2 (66.7%)
**Percentage reduction in hospitalizations**	100.0%	100.0%
**OCS dependency**		
***Baseline***		
OCS-dependent patients, n/N ^a^ (%)	7/38 (18.4%)	5/24 (20.8%)
***1-year FUP***		
OCS-dependent patients, n/N ^a^ (%)	6/41 (14.6%)	4/27 (14.8%)
**Daily dose of OCS (mg); N ^a^**	7	5
***Baseline***		
Median (IQR)	20 (7.5, 30.3)	10 (10.0, 15.0)
***1-year FUP***		
Median (IQR)	5 (0.0, 7.5)	0 (0.0, 5.8)
**Patients achieving OCS dose reduction ≥ 50%, n (%) ^b^**	5 (71.4%)	3 (60.0%)
**Patients achieving complete OCS withdrawal, n (%)**	3 (42.9%)	3 (60.0%)
**ACT score**		
***Baseline***		
ACT score, mean (SD)	14.1 (4.8)	15.1 (6.3)
Patients with ACT score < 20, n/N ^a^ (%)	30/35 (85.7%)	18/26 (69.2%)
***1-year FUP***		
ACT score, mean (SD)	22.2 (3.8)	20.4 (4.3)
Patients with ACT score < 20, n/N ^a^ (%)	5/29 (17.2%)	8/21 (38.1%)
**Increase in ACT score, mean (SD)**	7.5 (6.6)	4.9 (5.5)
**Patients with ACT increase ≥ 3, n/N ^a^ (%)**	19/25 (76.0%)	13/20 (65.0%)
**Lung function**		
***Baseline;* N ^a^**	41	26
Pre-BD FEV_1_ (% predicted), mean (SD)	54.3 (14.2)	60.0 (16.6)
Patients with pre-BD FEV_1_ < 80%, n (%)	38 (92.7%)	23 (88.5%)
***1-year FUP;* N ^a^**	30	17
Pre-BD FEV_1_ (% predicted), mean (SD)	71.5 (16.9)	73.6 (20.1)
Patients with pre-BD FEV_1_ < 80%, n (%)	21 (70.0%)	11 (64.7%)
***Baseline;* N ^a^**	41	26
Pre-BD FEV_1_ (mL), mean (SD)	1439.0 (452.8)	1919.2 (744.4)
***1-year FUP;* N ^a^**	30	17
Pre-BD FEV_1_ (mL), mean (SD)	1943.7 (722.9)	2320.0 (812.5)
**Increase in pre-BD FEV_1_ (mL), mean (SD)**	454.0 (462.5)	311.8 (353.6)
**Patients with pre-BD FEV_1_ increment ≥ 100 mL, n (%)**	21/30 (70.0%)	11/17 (64.7%)
**Patients with pre-BD FEV_1_ increment ≥ 230 mL, n (%)**	18/30 (60.0%)	8/17 (47.1%)
**Patients with pre-BD FEV_1_ increment ≥ 500 mL, n (%)**	12/30 (40.0%)	6/17 (35.3%)
**FeNO (ppb); N ^a^**	29	19
***Baseline***		
Median (IQR)	38 (19.4, 67.0)	39 (19.4, 63.9)
***1-year FUP***		
Median (IQR)	37 (16.8, 76.3)	52 (35.8, 63.5)

All values were calculated based on the total of patients with available data (excluding missing values). ^a^ Patients with available data. ^b^ Proportion of patients with 1 or more exacerbations at baseline who achieved a decrease in the frequency of exacerbations at 1-year FUP. ^c,d^ Calculations were made using the total number of patients with ED visits or hospitalizations, respectively. ACT, asthma control test; BEC, blood eosinophil count; ED, emergency department; FeNO, fraction of exhaled nitric oxide; FEV_1_, forced expiratory volume in the first second; FUP, follow-up; pre-BD, PAO, persistent airflow obstruction; pre-bronchodilator; SD, standard deviation; SMK, smoker.

## Data Availability

The datasets used and analyzed during the current study may be obtained in accordance with AstraZeneca’s data sharing policy, described at https://www.astrazenecaclinicaltrials.com/our-transparency-commitments/ (accessed on 9 April 2024).

## Supplementary Materials

The following supporting information can be downloaded at https://www.mdpi.com/article/10.3390/jcm14227900/s1. Table S1: Baseline sociodemographic and clinical characteristics of PAO+ patients classified by smoking habit (N = 68).

## Investigators ORBE II Study Group and Site

Alicia Padilla Galo, José Luis Velasco Garrido, Álvaro Martínez Mesa, Alberto Levy Naón, Isabel Moya Carmona and Carmen Cañete Luque (Hospital Virgen de la Victoria, Málaga); Borja Valencia Azcona (Hospital Costa del Sol, Málaga); Ismael García Moguel and Rocío Díaz Campos (Hospital Universitario 12 de Octubre, Madrid); Pilar Ausín and Eugenia Navarrete (Hospital del Mar, Barcelona); Luis Carazo Fernández (Complejo Asistencial Universitario de León, León); Eva Martínez Moragón (Hospital Universitario Doctor Peset, Valencia); Carlos Martínez Rivera (Hospital Universitario Germans Trias i Pujol, Barcelona); Francisco Casas Maldonado and Luis F. Cassini Gómez de Cádiz (Hospital San Cecilio, Granada); Rubén Andújar Espinosa (Hospital Clínico Universitario Virgen de la Arrixaca, Murcia); Elisabeth Vera Solsona and Marta Forner Vicente (Hospital Universitario Miguel Servet, Zaragoza); Andrea Trisán Alonso (Hospital Universitario Puerta de Hierro, Madrid); Fernando Sánchez-Toril López (Hospital Arnau de Vilanova, Valencia); Marcela Valverde Monge and Erwin Javier Pinillos (Hospital Universitario Fundación Jiménez Díaz, Madrid); Marina Blanco Aparicio (Complejo Hospitalario Universitario A Coruña, La Coruña); Marta Palop Cervera (Hospital de Sagunto, Valencia).

## Abbreviations

ACT	Asthma Control Test
CRSwNP	chronic rhinosinusitis with nasal polyps
FeNO,	fraction of exhaled nitric oxide
FEV_1_	forced expiratory volume in 1 s
IQR	interquartile range
OCS	oral corticosteroid
PAO	persistent airflow obstruction
SA	severe asthma
SD	standard deviation
SEA	severe eosinophilic asthma
SMK	smoking habit
