# Supplementary material for: ORBE II Study: Clinical Characteristics and Outcomes After Treatment with Benralizumab According to Airflow Obstruction Status and Smoking Habit
Source: J Clin Med. 2025 Nov 7;14(22):7900. doi: 10.3390/jcm14227900 (PMC12653045; doi:10.3390/jcm14227900)
Supplement: Supplementary file 1 [file jcm-14-07900-s001.zip › jcm-3916955-supplementary.pdf]

Supplementary Table S1. Baseline sociodemographic and clinical characteristics of PAO+ patients classified by smoking habit (N = 68).#

Variables	PAO+ SMK- n = 41	PAO+ SMK+ n = 27
**Sex, n (%)**		
Female	31 (75.6%)	11 (40.7%)
Male	10 (24.4%)	16 (59.3%)
**Age (years)**		
Mean (SD)	55.7 (14.5)	54.7 (11.4)
**BMI (Kg/m²); N[a]**	41	27
Mean (SD)	28.2 (6.2)	28.1 (7.0)
Obese[b], n (%)	13 (31.7%)	9 (33.3%)
**Age at asthma onset (years); N[a]**	31	20
Mean (SD)	32.8 (20.5)	31.4 (14.0)
**Asthma duration (years)[c]; N[a]**	31	20
Mean (SD)	20.8 (13.4)	20.3 (15.7)
**Allergic asthma, n (%)**	16 (39.0%)	7 (25.9%)
**Smoking history, n (%); N[a]**	41	27
Never smoker	41 (100.0%)	0 (0.0%)
Former smoker	0 (0.0%)	24 (88.9%)
Smoker	0 (0.0%)	3 (11.1%)
**Cigarette pack-year, n; N[a]**	0	57
Median (IQR)	NA	10 (5.8, 14.3)
**Comorbidities, n (%)[d]**	34 (82.9%)	26 (96.3%)
CRSwNP	12 (29.3%)	10 (37.0%)
COPD	0 (0.0%)	4 (14.8%)
Bronchiectasis	0 (0.0%)	4 (14.8%)
GERD	7 (17.1%)	7 (25.9%)
Osteoporosis	5 (12.2%)	2 (7.4%)
OSAS	4 (9.8%)	4 (14.8%)
HBP	9 (22.0%)	3 (11.1%)
Diabetes	5 (12.2%)	2 (7.4%)
Depression	4 (9.8%)	2 (7.4%)
Cataracts	2 (4.9%)	0 (0.0%)
**Patients with prior biologic treatment, n (%)[d]**		
Omalizumab	5 (12.2%)	4 (14.8%)
Mepolizumab[d]	5 (12.2%)	7 (25.9%)
Reslizumab[e]	1 (2.4%)	1 (3.7%)
Patients with no prior biologic treatment	31 (75.6%)	17 (63.0%)
**Peripheral BEC (cell/µL): N[a]**	39	26
Median (IQR)	540 (250.0, 765.0)	300 (125.0, 690.0)
**Total serum IgE concentration, (IU/mL); N[a]**	33	19
Median (IQR)	230 (59.0, 457.0)	274 (102.0, 393.5)
**FeNO (ppb); N[a]**	52	36
Median (IQR)	38 (19.4, 67.0)	39 (19.4, 63.9)
**OCS-dependency**		
OCS-dependent patients, n/N[a] (%)	7/38 (18.4%)	5/24 (20.8%)
**Daily dose of OCS (mg); N[a]**	7	5
Median (IQR)	20 (7.5, 30.3)	10 (10.0, 15.0)
Patients with daily OCS dose ≥5 mg, n (%)	7 (100.0%)	5 (100.0%)
**Severe exacerbations; N[a]**	41	27
Patients with severe exacerbations, n (%)	33 (80.5%)	22 (81.5%)
Severe exacerbations, mean (SD)	2.9 (2.8)	2.0 (1.4)
**ED visits; N[a]**	41	27
Patients with ED visits, n (%)	14 (34.1%)	7 (25.9%)
ED visits, mean (SD)	0.7 (1.7)	0.4 (0.8)
**Hospitalizations; N[a]**	41	27
Patients with hospitalizations, n (%)	32 (78.0%)	24 (88.9%)
Hospitalizations, mean (SD)	0.4 (0.9)	0.1 (0.3)
**Asthma control; N[a]**	35	26
ACT score, mean (SD)	14.1 (4.8)	15.1 (6.3)
Patients with ACT score <20, n (%)	30 (85.7%)	18 (69.2%)
**Lung function; N[a]**	41	26
Pre-BD FEV_1 (mL), mean (SD)	1439.0 (452.8)	1919.2 (744.4)
Pre-BD FEV_1 (% predicted), mean (SD)	54.3 (14.2)	60.0 (16.6)
Patients with pre-BD FEV_1 <80%, n/ N[a] (%)	38/41 (92.7%)	23/26 (88.5%)

All values were calculated based on the total of patients with available data (excluding missing values). Proportion of patients within each subgroup were calculated over the total of patients with available PAO and SMK data. Airflow obstruction status was defined as persistent (PAO+) if baseline post-BD FEV_1/FVC index was <0.7. Patients were also classified regarding their smoking habit at baseline into smokers (former/current) (SMK+) or non-smokers (SMK-).

[a] Patients with available data.

[b] BMI ≥30 Kg/m2

[c] Time in years since first asthma symptoms occurred.

[d] Multiple response variable.

ACT, asthma control test; BEC, blood eosinophil count; BMI, body mass index; COPD, Chronic obstructive pulmonary disease; CRSwNP, chronic rhinosinusitis with nasal polyposis; ED, emergency department; FeNO, fraction of exhaled nitric oxide; FEV_1, forced expiratory volume in the first second; GERD, gastroesophageal reflux disease; HBP, high blood pressure; IQR, interquartile range; OCS, oral corticosteroids; OSAS, obstructive sleep apnea syndrome; PAO, persistent airflow obstruction, pre-BD, pre-bronchodilator; SD, standard deviation; SMK, smoker.
